# The role of ceus in the characterization of indeterminate focal liver lesions at second-level imaging methods (INFOLIL STUDY)

**DOI:** 10.1007/s11739-025-04210-z

**Published:** 2025-11-25

**Authors:** Rossella Loiacono, Andrea Boccatonda, Alice Brighenti, Valeria Tiraferri, Daniela Agostinelli, Livia Masi, Nicola Venturoli, Sofia Maria Bakken, Carla Serra

**Affiliations:** Interventional, Diagnostic and Therapeutic Ultrasound Unit, IRCCS, Azienda Ospedaliero-Universitaria Di Bologna, 40138 Bologna, Italy

**Keywords:** CEUS, Liver, HCC, Ultrasound, Focal liver lesion

## Abstract

**Background:**

Focal liver lesions cannot always be characterized with certainty by using CT, MRI, or PET. In these cases, the Radiologist or Nuclear Medicine specialist often recommends performing CEUS.

**Purpose:**

the main aim was to determine the accuracy of CEUS in characterizing (as benign or malignant) focal liver lesions for which CT, MRI, and/or PET have not provided conclusive results.

**Material and Methods:**

a retrospective study was conducted by enrolling patients referred to our ultrasound unit to undergo CEUS examination based on the recommendation of the radiologist to characterize a focal liver lesion identified by CT, MRI, or PET. The reference gold standard was the histological examination in cases where it was performed; otherwise, findings from radiological and clinical follow-up were considered.

**Results:**

A total of 109 patients were enrolled in the study. Of these, 11 (10.1%) were not included in the analysis. The remaining 98 patients underwent ultrasound and CEUS examinations. Regarding the diagnostic accuracy of CEUS to characterize the nature of focal liver lesions in comparison with the reference standard, the method was characterized by an area under the curve (AUC) of 0.92 (95% CI: 0.83–1.00). The sensitivity of CEUS was 88.9% (95% CI: 65.3%–98.6%) and the specificity was 97.5% (95% CI: 91.2%–99.7%). For lesions smaller than 1 cm in diameter, all lesions in this category were characterized as benign on CEUS. For lesions between 1 and 2 cm in size, the AUC reached 1.00, with both sensitivity and specificity at 100%, suggesting an optimal performance of CEUS for this category of lesions. Regarding lesions larger than 2 cm, the AUC was 0.96 (95% CI: 0.85–1.00). Sensitivity was 90.9% (95% CI: 58.7%–99.8%), and specificity was 100% (95% CI: 78.2%–100%).

**Conclusions:**

CEUS proves to be a valuable diagnostic tool in the characterization of focal liver lesions, improving clinical management with a less invasive approach. CEUS may be an integral part of the diagnostic pathway for patients with indeterminate focal liver lesions.

**Key results**

- CEUS demonstrated an overall AUC of 0.92 in characterizing indeterminate focal liver lesions.
- Sensitivity was 88.9%, with specificity of 97.5%.
- For lesions measuring 1–2 cm, CEUS achieved 100% sensitivity and specificity.

## Introduction

Ultrasound has traditionally been considered a first-line imaging technique, with magnetic resonance imaging (MRI), computed tomography (CT), and positron emission tomography (PET) reserved for resolving uncertainties regarding the characterization of lesions detected on B-mode. However, the advent of contrast-enhanced ultrasound (CEUS) has elevated ultrasound to a second-level imaging method, on par with other contrast-enhanced techniques [1]. CEUS can characterize lesions identified in B-mode by studying their vascular dynamics [2]. CEUS is recommended for the characterization of incidental focal liver lesions in non-cirrhotic patients with negative oncological history, as well as in cases where contrast-enhanced CT or MRI are inconclusive or contraindicated [3]. Moreover, CEUS can also be applied in oncological patients, where it contributes to the characterization of newly detected or indeterminate liver lesions [3]. CEUS is also suggested in those cases where biopsy results have been inconclusive [4]. In cirrhotic patients, CEUS is used to characterize focal liver lesions through the LI-RADS classification, especially in those with contraindications to contrast-enhanced CT or MRI [4].

In cases of liver lesions on the cirrhotic liver with inconclusive diagnoses through contrast-enhanced CT or MRI (LR-3, LR-4, LR-M lesions), CEUS can be used as a non-invasive diagnostic method if the lesions are visible on B-mode [4]. In our daily clinical practice, we are increasingly encountering liver lesions that cannot always be characterized with certainty by using CT, MRI, or PET. In these cases, the Radiologist or Nuclear Medicine specialist often recommends performing CEUS to further characterize these lesions.

The main aim of our study was to determine the accuracy of CEUS in characterizing (as benign or malignant) focal liver lesions for which CT, MRI, and/or PET have not provided conclusive results. The study endpoint was to evaluate the diagnostic accuracy, specificity and sensitivity, positive and negative predictive values of CEUS in characterizing both benign and malignant focal hepatic lesions. We further performed a diagnostic accuracy sub-analysis based on lesion size.

## Materials and methods

### Study design

A retrospective diagnostic study was conducted from February 1, 2022, to February 28, 2024. The reference gold standard was the histological examination in cases where it was performed; in other cases, a 6-month clinical and radiological follow-up was considered. Definitive diagnosis was established by histopathology when available (percutaneous biopsy or surgical specimen; *n* = 8). In the absence of tissue, a ≥ 6-month imaging follow-up served as the reference (*n* = 46), defined as: (1) benign when the lesion showed stability or resolution, or developed unequivocal benign features on contrast-enhanced imaging; (2) malignant when it demonstrated unequivocal progression (e.g., new/enlarging lesion or ≥ 20% diameter increase) and/or malignant enhancement features on subsequent contrast-enhanced CT/MRI/CEUS, or when cancer-directed therapy was initiated based on concordant imaging. When imaging remained indeterminate after follow-up, the final diagnosis was assigned by multidisciplinary consensus at the 6-month review (radiology, hepatology, oncology, and surgery; *n* = 44). To avoid double-counting when both clinical and radiological follow-up were available, each lesion was assigned a single reference category by prespecified hierarchy (histology > imaging follow-up > multidisciplinary consensus).

### Study population

Patients were enrolled at the Departmental Program of Interventional, Diagnostic, and Therapeutic Ultrasound, IRCCS Policlinico Sant’Orsola-Malpighi, Bologna (Italy), based on a radiologist’s indication for CEUS to characterize a focal liver lesion previously detected by CT, MRI, or PET and not conclusively diagnosed. Inclusion criteria were:

- age ≥ 18 years;
- presence of at least one focal liver lesion requiring characterization after non-diagnostic CT, MRI, and/or PET;
- availability of a reference standard for definitive diagnosis (histology, imaging follow-up, or multidisciplinary consensus at 6-month follow-up;
- written informed consent obtained.

Patients with or without underlying hepatic or extra-hepatic disease (e.g., hepatopathic, oncological, or other clinical conditions) were considered eligible. No dimensional limits were applied to the liver lesions considered for analysis. Patients were excluded from the analysis in the following circumstances: when CEUS was not performed, when they were lost to follow-up, or when death occurred before completing six months of observation.

### Ultrasound and CEUS protocol

The ultrasound and CEUS examinations were performed using a convex probe with a frequency range of 1.0–5.0 MHz; the dynamic range was set to 42, and the mechanical index was low [4]. The CEUS examination was performed according to the methods recognized by the relevant guidelines [5, 6]. The diameter of the venous line was 20 gauge or larger to minimize the destruction of microbubbles as they passed through the cannula, with the length being kept as short as possible. An ultrasound contrast agent (UCA) (SonoVue, Bracco, Milan, Italy), which acts as a pure intravascular agent consisting of micro-bubbles (1–7 micron) that contain sulfur hexafluoride encapsulated by a phospholipid shell, was employed. The injection bolus for SonoVue was delivered at approximately 1–2 mL/s. Immediately following the injection of the contrast medium, a bolus of (5-) 10 mL of saline was administered to flush the line at approximately 2 mL/s. The recommended dose of SonoVue for the detection and characterization of liver lesions was 2.4 mL [5, 6].

On CEUS examinations, the enhancement of liver lesions is assessed dynamically through three main vascular phases. The arterial phase starts approximately 10–20 s after contrast administration and lasts up to 30–45 s; in this interval hypervascular lesions, such as hepatocellular carcinoma, some metastases, or focal nodular hyperplasia, may show an early increase in enhancement compared with the surrounding parenchyma. The portal venous phase follows, extending roughly from 30–45 s to about 120 s, when the liver parenchyma enhances via portal inflow and differences between benign and malignant lesions begin to emerge. The late (parenchymal) phase extends from about 120 s up to 4–6 min, when the liver remains homogeneously enhanced, whereas many malignant lesions progressively lose contrast and appear hypoenhancing. The diagnostic characterization of focal liver lesions is therefore based on the recognition of these enhancement patterns across the different phases. Benign lesions typically maintain enhancement in the late phase, sometimes with specific features such as peripheral nodular enhancement with centripetal fill-in in hemangiomas, or homogeneous persistent enhancement in focal nodular hyperplasia. Conversely, malignant lesions are usually characterized by early or late washout, with the timing and intensity of washout providing additional diagnostic clues: early and marked washout being highly suggestive of metastases or cholangiocarcinoma, while late and mild washout is more typical of hepatocellular carcinoma.

In some patients, more than one focal liver lesion was present. In these cases, the ultrasound and CEUS evaluation focused exclusively on the lesion(s) reported by the radiologist as requiring further characterization.

Since the retrospective study design, the single examination was performed by a single physician. All the physicians who performed CEUS in the study were characterized by over ten years of expertise in liver and abdominal ultrasonography and CEUS.

### Statistical analysis

The demographic and clinical characteristics of the patients are presented as frequencies and percentages for categorical variables and as means ± standard deviation or median and range for continuous variables. Statistical analysis was performed using SPSS software (IBM SPSS Statistics Version 25, Inc., Chicago, IL, USA). To evaluate the diagnostic accuracy of CEUS, a Receiver Operating Characteristic (ROC) curve was generated, and the Area Under the Curve (AUC) was calculated using SPSS software. Additionally, sensitivity, specificity, positive likelihood ratio (PLR), negative likelihood ratio (NLR), and diagnostic odds ratio (DOR) were calculated by constructing 2 × 2 contingency tables, determining true positives, false positives, false negatives, and true negatives.

## Results

**Fig. 1:**
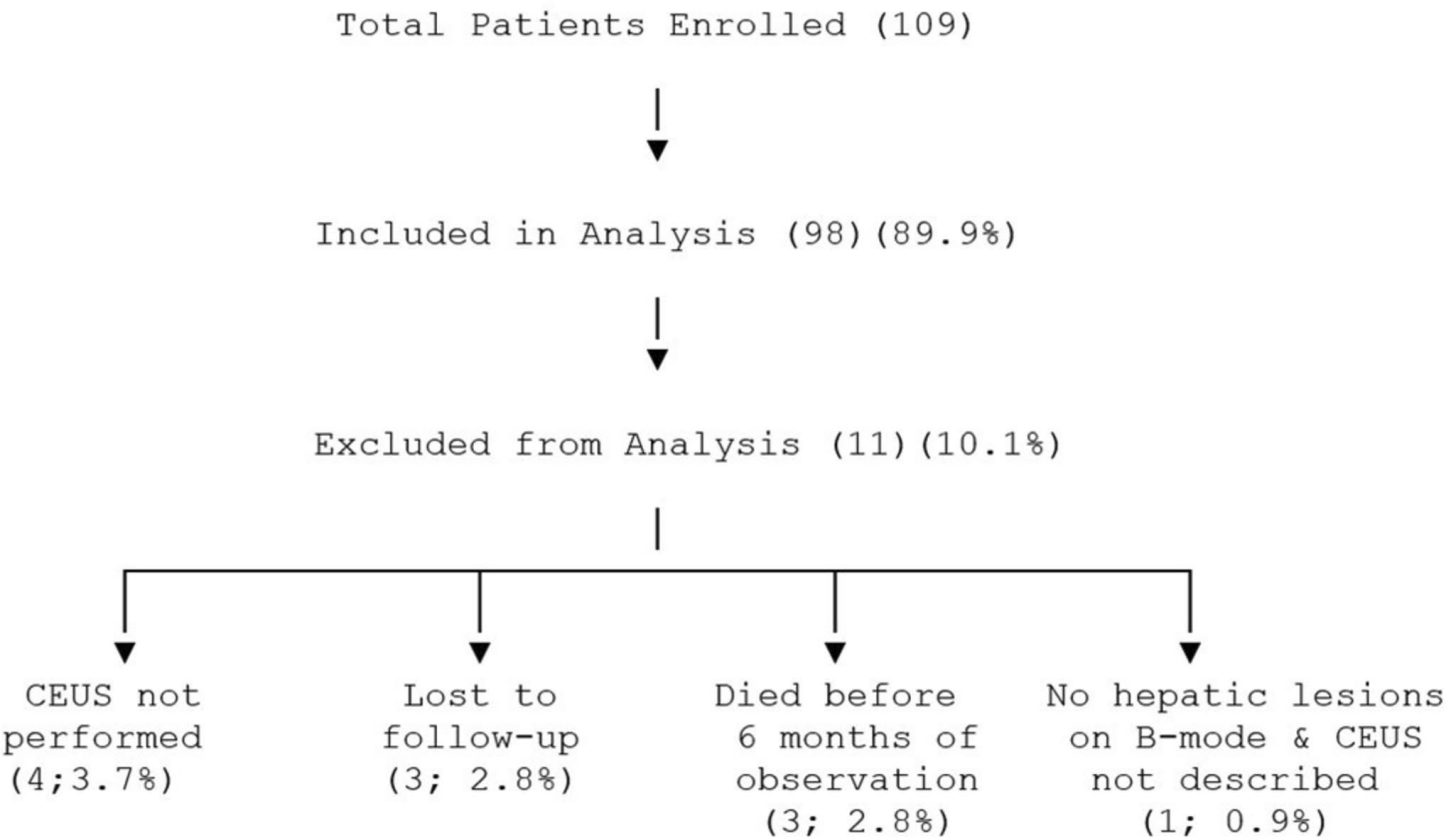
Flow-diagram of the study

A total of 109 patients were enrolled in the study. Of these, 11 (10.1%) were not included in the analysis (Fig. 1). Patients were excluded from the subsequent ultrasound and CEUS examinations in the following circumstances: when CEUS was not performed (*n* = 4; 3.7%), when they were lost to follow-up (*n* = 3; 2.8%), or when death occurred before completing six months of observation (*n* = 3; 2.8%). In addition, one patient (*n* = 1; 0.9%) was excluded because no liver lesions were detected on B-mode ultrasound and CEUS was not described. Of the remaining 98 patients, 36 (36.7%) were female and 62 (63.3%) were male. The mean age was 55.1 years ± 13.4 (SD). The lesions being examined occurred on a cirrhotic liver in 5 cases (5.1%); in 3 cases (3.1%) patients had intrahepatic primary neoplasia, in 72 cases (73.5%) patients had a positive history of extrahepatic primary neoplasia, and in 12 cases (12.2%) patients had other pathologies.

CEUS was performed following a non-diagnostic CT in 66 cases (67.3%), in 16 cases (16.3%) following a non-diagnostic MRI, and in 16 cases (16.3%) following a non-conclusive PET scan.

Of the 98 lesions, 10 (12.5%) were investigated by using second-level techniques without contrast medium, and 70 (87.5%) were investigated with contrast medium. The remaining 18 lesions were studied with PET or MR cholangiography. Each patient had undergone only one second-level imaging examination (CT, MRI, or PET) before referral for CEUS; no cases with multiple prior imaging modalities were included. Main characteristics of the lesions were described in Tables 1 and 2.

**Table 1:** Demographic data and baseline characteristics

Category	Number of cases	Percentage (%)
Gender		
Female	36	36.7
Male	62	63.3
Basal Characteristics		
Cirrhotic liver	5	5.1
Intrahepatic primary neoplasm	3	3.1
Extrahepatic primary neoplasm	72	73.5
Healthy liver	93	94.8
Indication for CEUS		
Non-diagnostic CT	66	67.3
Non-diagnostic MRI	16	16.3
Non-diagnostic PET	16	16.3
Investigation Method		
Without contrast agent	10	10.2
With contrast agent	70	71.4
Cholangiography-MRI or PET	18	18.4

CEUS, contrast-enhanced ultrasound; CT, computed tomography; MRI; magnetic resonance imaging; PET, positron emission tomography

**Table 2:** Lesion Characteristics on Second-Level Imaging. Abbreviations: PET, positron emission tomography; HCC, hepatocellular carcinoma; FNH, focal nodular hyperplasia

Lesion Characteristics on Second-Level Imaging	*n* (%)
Non-avid on PET	1 (1%)
Avid on PET	12 (12.2%)
Hypodense in arterial phase	2 (2%)
Hyperdense in arterial phase	9 (9.2%)
Isodense	1 (1%)
Hyperdense in venous phase	1 (1%)
Constantly hypodense	4 (4.1%)
Hyperdense	1 (1%)
Hyperintense on T2 Hyperintense on T2	1 (1%)
Hypodense	25 (25.5%)
Hypodense with late homogeneity	5 (5.1%)
Uncharacterized	1 (1%)
Other	35 (35.7%)

Second-Level Imaging Characteristics	Number of cases	Percentage (%)
Lesions Size		
< 1 cm	26	26.53
1–2 cm	44	44.9
> 2 cm	12	12.24
Not specified	11	11.22
Number of Lesions		
Single	72	73.5
Two	14	14.3
Three or more	12	12.2
Wash-out		
With washout	69	89.6
Without washout	8	10.4
Presumptive Diagnosis		
Not clear – not formulated	50	51.0
Metastasis	7	7.1
HCC	1	1.0
Benign lesion (hemangioma, FNH, cyst, area of fatty sparing)	35	35.7
Other (non-malignant)	5	5.1

### B-mode ultrasound characteristics of the lesions

The B-mode characteristics of the lesions were: in 50 cases (51.0%) the lesion was solitary, in 12 cases (12.2%) two lesions were visualized, in 10 cases (10.2%) the lesions numbered more than two, and in 26 cases (26.6%) the lesion to be investigated was not visible in B-mode. Dimensions were specified for 68 cases: 9 lesions had a diameter < 1 cm (13.2%), 33 lesions (48.5%) were between 1 and 2 cm, and 26 lesions (38.2%) were > 2 cm (Table 3). In our center, B-mode ultrasound is systematically performed before CEUS, and a preliminary presumptive, but not definitive, diagnosis is generally formulated, which may subsequently be confirmed or modified by contrast-enhanced evaluation. Notably, all patients remained eligible for inclusion, since the criterion of lesion indeterminacy was always defined in advance, based on the non-diagnostic findings of prior CT, MRI, or PET performed outside our institution. In most cases (78/98 patients), the reporting physician formulated a presumptive diagnosis before performing the CEUS: in 41 cases (52.6%), the diagnosis was unclear or no diagnosis was provided, in 3 cases (3.8%) secondary malignancy from an extrahepatic primary was suspected, in 30 cases (38.5%) the lesion was presumed to be benign, in 1 case (1.3%) a regenerating nodule was suspected, and in 3 cases (3.8%) other diagnoses were considered (Table 3).

**Table 3:** B-mode Ultrasound Characteristics of the Lesions

B-mode Characteristics	Number of cases	Percentage (%)
Lesions size		
< 1 cm	9	9.18
1–2 cm	33	33.67
> 2 cm	26	26.53
Not specified – not visualized	30.61	30.61
Appearance		
Hyperechoic	27	38.6
Isoechoic	2	2.9
Hypoechoic	20	28.6
Anechoic	10	14.3
Heterogeneous	2	2.9
Other	9	12.9
Presumptive Diagnosis		
Not clear – not formulated	41	52.6
Metastasis	3	3.8
Regenerative nodule	1	1.3
Benign lesion (hemangioma, FNH, cyst, area of fatty sparing)	30	38.5
Other (non-malignant)	3	3.8

### CEUS examination

In the arterial phase, the vascular behavior of the lesions was: 12 lesions (12.2%) were avascular, 3 lesions (3.1%) were hypovascular, 16 lesions (16.3%) were isovascular, 19 lesions (19.4%) were hypervascular, 2 lesions (2.0%) showed peripheral enhancement, 13 lesions (13.3%) exhibited globular peripheral enhancement, 5 lesions (5.1%) showed other characteristics, and 28 lesions (28.6%) were not visualized in the arterial phase. Of the 98 lesions evaluated with CEUS, 79 cases (80.6%) showed no washout in the lesion and/or the hepatic parenchyma (in cases where the lesion was not visualized). In contrast, 19 cases (19.4%) exhibited washout in the studied lesion and/or other areas of the hepatic parenchyma (Table 4).

**Table 4:** CEUS Characteristics of the focal liver lesions

CEUS Characteristics	Behavior	Number of cases	Percentage (%)
Arterial Phase	No enhancement (avascular)	12	12.2
	Hypoenhancement	3	3.1
	Isoenhancement	16	16.3
	Hyperenhancement	19	19.4
	Peripheral globular enhancement	13	13.3
	Peripheral enhancement	2	2.0
	Other	5	5.1
	Not visualized	28	28.6
Venous Phase	No enhancement (avascular)	12	20.0
	Hypoenhancement	11	18.3
	Isoenhancement	27	45.0
	Hyperenhancement	3	5.0
	Not specified	7	11.7
Late Phase	No enhancement (avascular)	12	17.1
	Hypoenhancement	19	27.1
	Isoenhancement	29	41.4
	Hyperenhancement	3	4.3
	Avascular in central area	7	10.0
Wash-out	Absent	79	80.6
	Present	19	19.4

Specifically, the degree of enhancement observed in the lesion during the venous phase was described for 60 lesions: 12 (20.0%) were avascular, 11 (18.3%) were hypovascular, 27 (45.0%) were isovascular, 3 (5.0%) remained hypervascular, and 7 (11.7%) were not visualized. Moreover, the degree of enhancement of the lesion during the late phase was described for 70 lesions: 12 (17.1%) were avascular, 19 (27.1%) were hypovascular, 29 (41.4%) were isovascular in the hepatic parenchyma, 3 (4.3%) remained hypervascular, and 7 (10.0%) were described as avascular in the central portions.

For all cases, it was specified whether the lesion was characterized as benign or malignant based on the outcome of the multiparametric ultrasound exam: in 79 cases (80.6%) CEUS concluded the lesion was benign, and in 19 cases (19.4%) it concluded the lesion was malignant (Table 5).

**Table 5:** Contrast-enhanced ultrasound-based diagnosis of focal liver lesions and final classification of their nature (benign or malignant)

Final classification of the nature of the lesion		
Benign	79	80.6
Malignant	19	19.4
Total	98	100
Specific diagnosis (subtype) of the lesion on CEUS	Number of cases	Percentage(%)
Not clearly expressed	8	8.1
Metastasis	15	15.3
Hepatocellular carcinoma	3	3.0
Benign lesion (cyst, hemangioma, focal nodular hyperplasia, area of different fat distribution)	49	50.0
Regenerative nodule	3	3.0
Other benign lesions	20	20.4

^a^Bulging of the hepatic parenchyma was observed in 1 case (1.0%), peripheral bile duct ectasia was detected in 1 case (1.0%), hepatic abscess in 1 case (1.0%), and focal sclerosing cholangitis in 1 case (1.0%); in 16 cases (16.3%) the lesions were not visible on CEUS (with no wash-out) and were therefore considered benign

Regarding the specific diagnosis and the sub-type of lesion, in 8 cases (8.2%), the diagnosis was unclear or not expressed, in 15 cases (15.3%) the CEUS indicated the lesion as secondary, in 3 cases (3.1%) as HCC, in 49 cases (50.0%) the CEUS diagnosed a benign lesion (angioma, FNH, area with altered fat distribution, cyst), in 3 cases (3.1%) a regenerating nodule was identified, and in 20 cases (20.4%) a lesion of other benign nature was diagnosed. These last were categorized as follows: in 1 case, the lesion was identified as deformation of normal hepatic parenchyma; in 16 cases, the lesion was not visualized on CEUS but no areas of the early washout were documented, leading the ultrasound operator to conclude the lesion was benign; in 1 case, the diagnosis was biliary duct ectasia, in 1 case a hepatic abscess, and 1 case focal sclerosing cholangitis/previous cholangitic change.

### Follow-up findings

Particularly, one (1.0%) patient underwent an excisional biopsy of the lesion; 2 patients (2.0%), whose lesions were found during screening for solid organ donation and solid organ transplant list placement, completed the corresponding surgical procedure; 3 patients (3.0%) underwent radical surgery for extrahepatic primary neoplasia after excluding secondary nature of the lesion; 2 patients (2.0%) received locoregional treatments after CEUS characterized the lesions as neoplastic; 7 patients (7.1%) had the outcome of CEUS compared with histological analysis of the lesion after percutaneous biopsy; 1 patient (1.0%) was started on chemotherapy after a CEUS diagnosis of the malignant lesion; 40 patients (40.8%) were followed up clinically; 68 patients (69.4%) were followed up radiologically (Table 6. Suppl. Material). By avoiding double-counting when both clinical and radiological follow-up were available, across the 98 analyzed lesions, the reference standard was histology in 8 cases, ≥ 6-month imaging follow-up in 46 patients, and multidisciplinary consensus in 44 patients.

**Table 6:** Type of Intervention/Follow-up

Type of Intervention/Follow-up	Number of patients	Percentage (%)
Excisional biopsy	1	1.0
Percutaneous biopsy	7	7.1
Specific surgical pathway (screening for organ donation/transplant)	2	2.0
Radical surgery for extrahepatic primary neoplasm	3	3.0
Locoregional treatments (chemoembolization, thermoablation)	2	2.0
Initiation of chemotherapy	1	1.0
Clinical follow-up (specialist visits)	40	40.8
Radiological follow-up (CT, MRI, PET)	68	69.3

Overall, the global follow-up concluded that 80 lesions (81.6%) were benign, and 18 lesions (18.4%) were malignant. Specifically: 15 lesions (15.3%) were characterized as secondary from extrahepatic primaries, 3 lesions (3.1%) as HCC, 47 lesions (48%) as benign (angioma, FNH, area with altered fat distribution, cyst), 3 lesions (3.1%) as regenerating nodules, and 30 lesions (30.6%) were of other benign nature. Specifically, of these, 22 were not reported on subsequent follow-up, 3 were characterized as areas of altered vascularization, 1 as deformation of the normal hepatic parenchyma, 1 as an abscess, 1 as focal sclerosing cholangitis/previous cholangitis change, 1 was reported as stationary without specifying the nature in subsequent radiological investigations, and 1 remained uncharacterized (Table 5).

### CEUS diagnostic accuracy

**Fig. 2:**
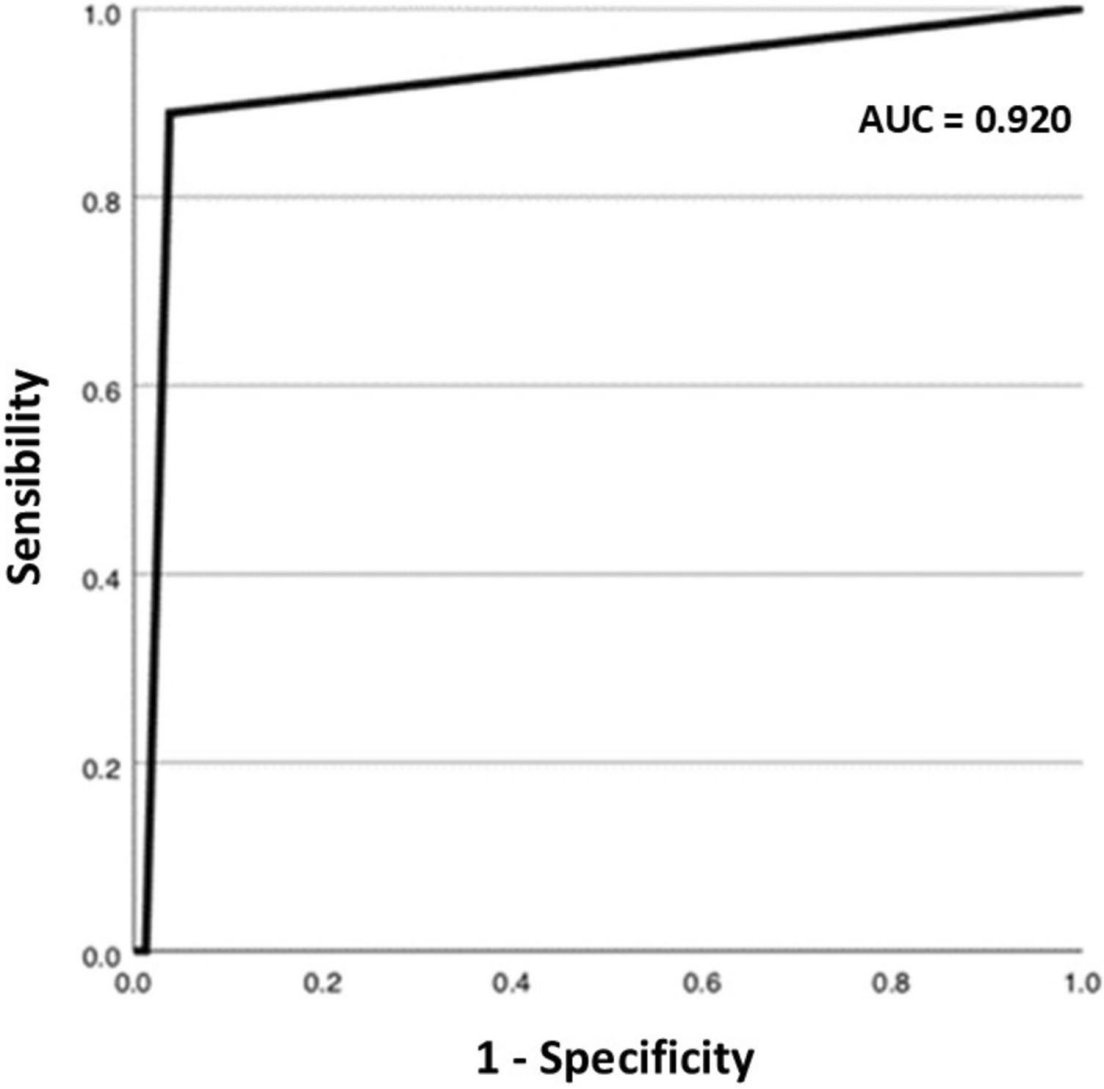
ROC curve regarding CEUS ability to detect focal liver lesions malignancy in comparison with the overall clinical follow-up

The follow-up results show that CEUS was conclusive in 92 cases (93.9%) and inconclusive in 6 cases (6.1%). In 92 cases (93.9%), the diagnosis made by CEUS agreed with the reference gold standard, while in 3 cases (3.1%) it was discordant. Regarding the CEUS ability to detect focal liver lesions malignancy in comparison with the overall clinical follow-up, the method was characterized by an AUC of 0.92 (95% CI: 0.83–1.00) (Fig. 2). The sensitivity of CEUS was 88.9% (95% CI: 65.3%–98.6%) and the specificity was 97.5% (95% CI: 91.2%–99.7%). A PLR of 35.1 (95% CI: 8.85–139.28), a NLR of 0.11 (95% CI: 0.03–0.42), and a DOR of 308.00 (95% CI: 40.34–2351.2) were found.

The diagnostic accuracy of CEUS was then analyzed according to the size of the lesions. For lesions smaller than 1 cm in diameter, it was not possible to calculate the AUC, sensitivity, or specificity, as all lesions in this category were characterized as benign on CEUS.

**Fig. 3:**
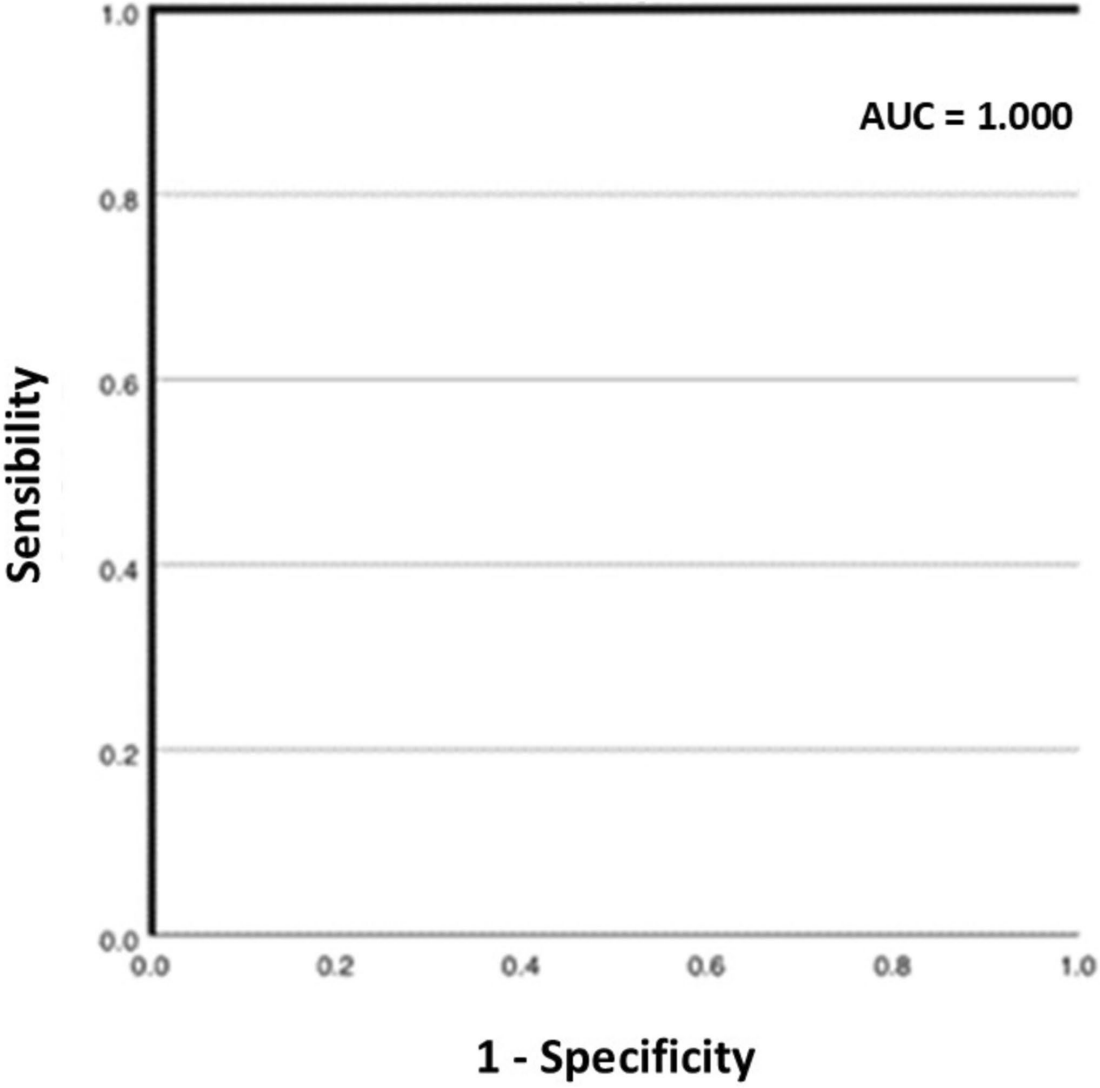
ROC curve regarding CEUS ability to detect focal liver lesions between 1 and 2 cm in size in comparison with the overall clinical follow-up

For lesions between 1 and 2 cm in size, diagnostic accuracy was even higher: the AUC reached 1.00, with both sensitivity and specificity at 100%, suggesting an optimal performance of CEUS for this category of lesions (Fig. 3). The PLR in this group was 53.2, while the NLR was very low, at 0.085, with a DOR of 627.00 (95% CI: 11.2–35,093.3).

**Fig. 4:**
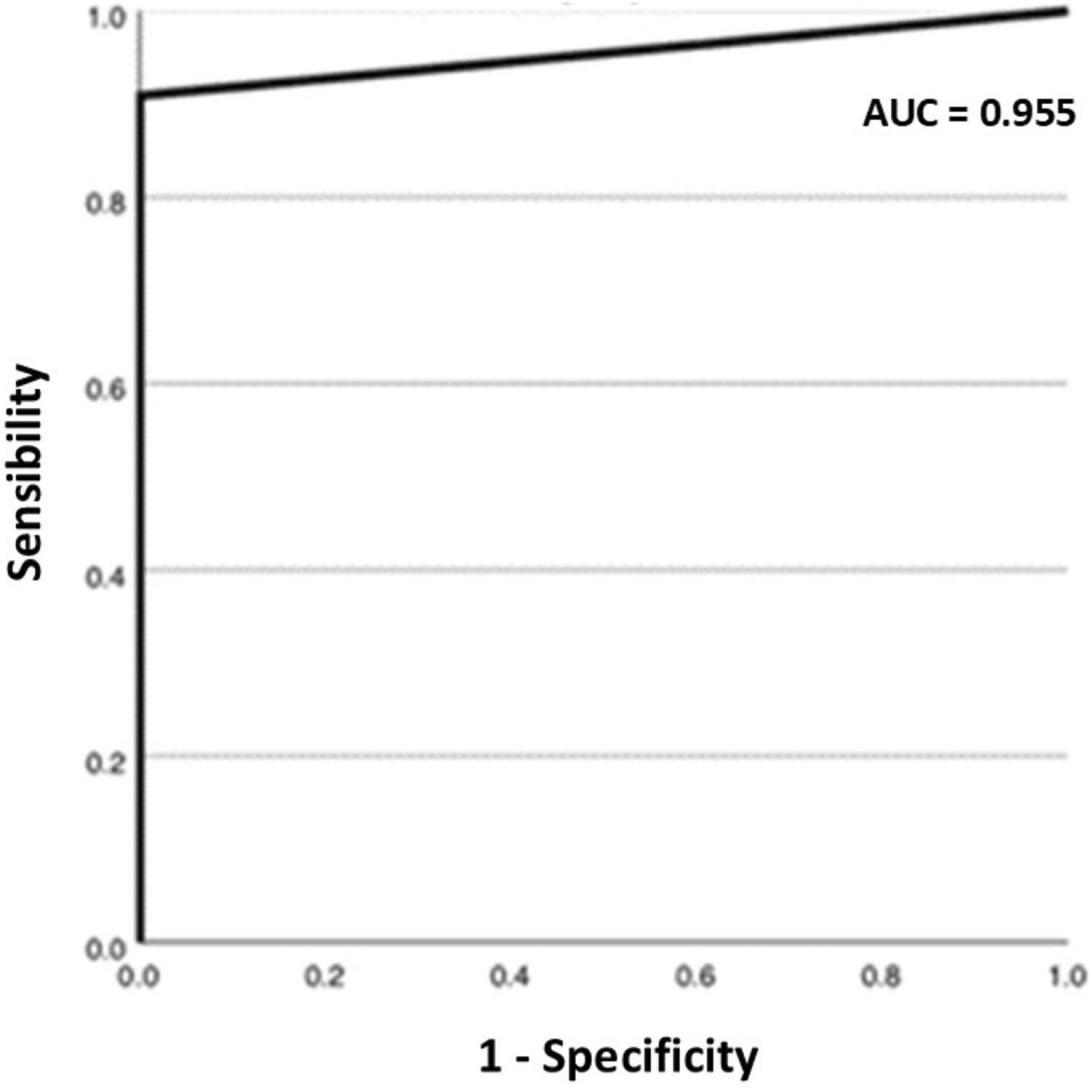
ROC curve regarding CEUS ability to detect focal liver lesions > 2 cm in size in comparison with the overall clinical follow-up

Finally, for lesions larger than 2 cm, the AUC was 0.96 (95% CI: 0.85–1.00) (Fig. 2), also demonstrating excellent accuracy. Sensitivity was 90.9% (95% CI: 58.7%–99.8%), and specificity was 100% (95% CI: 78.2%–100%) (Fig. 4). In this group, the PLR was 28.0, while the NLR was 0.13, with a DOR of 217.00 (95% CI: 8.04–5854.7).

## Discussion

In everyday clinical practice, it is increasingly common to encounter patients undergoing second-level imaging studies that failed to conclusively characterize focal liver lesions, particularly those of small size.

Our study demonstrated that CEUS has high diagnostic accuracy in characterizing indeterminate focal liver lesions, with an AUC of 0.92. This is comparable to that of MRI, as evidenced in a previous study by D’Onofrio et al. [7], which showed how CEUS is particularly useful for assessing lesion vascularity in real-time. The study concluded that CEUS and MRI with hepato-specific contrast agents are complementary techniques: the former for its speed and vascular characterization of malignant lesions, and the latter for a more detailed overall assessment of the liver parenchyma, with diagnostic accuracy of 91% for CEUS and 92% for MRI. In our sample, the sensitivity found was 88.9%, while specificity reached 97.5%. Previous studies, such as that of Quaia et al. [8], highlighted how CEUS significantly improves the characterization of liver lesions compared to CT, confirming our results. In the multicenter DEGUM trial [9], CEUS demonstrated a sensitivity of 95.3% and specificity of 83.7%, confirming the superiority of this technique over CT (sensitivity 90%, specificity 81.6%).

It was not possible to perform a statistical analysis for the 9 lesions smaller than 1 cm because all of them were classified as benign on CEUS. For lesions between 1 and 2 cm in size, the results were excellent: the AUC reached the maximum value of 1.00, with both sensitivity and specificity at 100%. In the case of lesions larger than 2 cm, CEUS accuracy remained high, with an AUC of 0.96, but a slight decrease was observed compared to smaller lesions. This could be attributed to the increased complexity of contrast-enhanced characteristics in larger lesions, as previously reported in other studies [10, 11].

In general, CEUS proved particularly effective in characterizing liver lesions smaller than 2 cm, where CT and MRI may have limitations. Previous studies have shown that CEUS accuracy in detecting liver metastases is comparable to that of contrast-enhanced CT and MRI [4], with Dietrich et al. [12] stating that CEUS is superior to CT and comparable to MRI in the differential diagnosis of focal liver lesions.

Presumptive diagnoses made by radiologists before CEUS showed a certain degree of diagnostic uncertainty, highlighted by the fact that no clear hypothesis was made in 51% of cases. However, CEUS provided significant diagnostic clarification, with 93.9% agreement with the gold standard, thus confirming its validity as an imaging method for the evaluation and characterization of focal liver lesions.

Finally, we note the clear contrast between the absence/presence of washout indicated by second-level imaging techniques (washout present in 89.6% of cases) and CEUS (washout present in 19.4% of cases). A possible explanation may be related to the different contrast agents used and their distinct characteristics and dynamics. Contrast agents used in CT and MRI diffuse into the endothelium, while the ultrasound contrast agent is purely intravascular during the late phase [13]. Other factors to consider include the ability to visualize the contrast behavior of lesions in real-time and throughout the vascular phases offered by CEUS, as well as the absence of a blinded comparison. The lack of washout on MRI is one of the main causes of missed characterization of liver lesions in this method [14]. Due to its real-time performance, CEUS is very sensitive in identifying washout areas, offering detailed information about the onset and intensity of washout, not only allowing for the characterization of a lesion as malignant but also enabling differentiation, for example, between HCC and ICC.

In only 3 cases, CEUS and follow-up findings were discordant. One case involves a patient with pancreatic adenocarcinoma who had undergone surgical treatment followed by adjuvant chemotherapy. She also had concomitant thrombosis of the middle hepatic vein. CEUS was performed following a CT scan that reported a 15 mm area adjacent to the thrombosis. The CEUS did not show the lesion or areas of pathological hepatic washout, so an MRI was recommended. However, the MRI was also inconclusive. After an initially inconclusive radiological follow-up and a progressive increase in CA 19.9 blood levels, the patient underwent diagnostic laparoscopy, during which the hepatic lesion was directly visualized and removed after converting to open surgery thus showing a neoplasm of biliopancreatic nature.

Another case involves a woman with breast cancer, who was referred for CEUS after a PET scan in January 2022 showing 2 hepatic lesions with FDG uptake. In February, the CEUS showed an area of pathological washout corresponding to one of the reported lesions, which was thus characterized as secondary metastasis. In May of the same year, after completing chemotherapy, the patient underwent a CT scan for reevaluation, which was negative for secondary hepatic lesions, as was another CT performed in October.

Finally, in the last case, the CEUS performed to characterize the hepatic lesion yielded inconclusive results, and subsequent radiological follow-ups did not show the lesion again.

CEUS has numerous advantages, including excellent diagnostic efficacy, speed of execution, and significant cost savings. CEUS offers accuracy comparable to MRI and superior to CT in the characterization of focal liver lesions. This efficacy is attributed to the ability to assess washout in real time [12]. Moreover, CEUS can reduce the need for invasive procedures, promoting a more conservative approach to managing indeterminate liver lesions. CEUS is highly operator-dependent, and the outcome also depends on the quality of the equipment used. In the case of lesions located in anatomically challenging positions, such as in obese patients or in the presence of significant meteorism, lesion visibility may be compromised. Other factors limiting the use of CEUS include significant comorbidities in patients (particularly hepatic steatosis and lack of cooperation) and the lack of panoramic coverage compared to other imaging techniques.

### Study limitations

It was conducted at a single center with highly skilled operators and advanced equipment, which may not reflect conditions in other imaging centers. Statistical analysis could not be performed for the sub-centimetric lesions category due to the benign diagnosis made by CEUS for all the lesions examined. The data regarding imaging methods, including B-mode ultrasound and CEUS evaluation, were collected from the reports only. Therefore, given the retrospective nature of the study, it was not possible to have each examination reviewed by multiple operators. Consequently, a single physician was responsible for reporting the ultrasound and CEUS examinations. Each CEUS exam was performed by a single operator, lacking a blinded comparison between different performing physicians. Additionally, our study has a retrospective nature, which introduces potential biases in patient selection and data collection. Comprehensive clinical data (e.g., comorbidities and the precise indication for second-level imaging) were not consistently retrievable from the retrospective report review. As a result, CEUS interpretations were not blinded to clinical context, which may limit reproducibility across settings; we therefore limited Table 1 to baseline features available in all patients and explicitly acknowledge this constraint.

## Conclusions

Our study highlighted the significant role of CEUS in characterizing indeterminate focal liver lesions detected by second-level imaging techniques. CEUS offers high diagnostic accuracy, with an overall AUC of 0.92, and excels particularly for lesions between 1 and 2 cm, where it achieved an AUC of 1.00 with both sensitivity and specificity at 100%. These results are especially relevant for patients with chronic liver disease or a history of cancer, as early diagnosis of hepatocellular carcinoma or liver metastases can have a decisive impact on prognosis and treatment options.

Although statistical analysis could not be performed for sub-centimetric lesions, the confirmation of benignity through patient follow-up suggests that CEUS may still provide valuable diagnostic information. This is particularly significant, given that 51% of the presumptive diagnoses made by radiologists before the exam did not offer a clear hypothesis, limiting therapeutic or follow-up decisions. The 93.9% agreement of CEUS with the gold standard emphasizes its validity as an imaging method for evaluating and characterizing focal liver lesions.

The technique is particularly relevant in the follow-up of cancer patients, with high sensitivity in identifying malignant lesions and good reliability in excluding disease, thus reducing diagnostic uncertainty and assisting oncologists in clinical management. Based on the results from our study, we recommend considering CEUS as an integral part of the diagnostic pathway for patients with indeterminate focal liver lesions, especially those with a history of neoplasia. Eventually, future research should focus on the development of new technologies and contrast agents that could further optimize the use of CEUS in liver lesion diagnosis.

## Summary statement

Contrast-enhanced ultrasound effectively characterizes indeterminate liver lesions, especially those between 1 and 2 cm, providing high diagnostic accuracy, sensitivity, and specificity, and offering a cost-effective alternative to other imaging techniques.

## Data Availability

Data are available upon request from authors.

## References

[1] Sporea I, Şirli R (2014) Is contrast enhanced ultrasound (CEUS) ready for use in daily practice for evaluation of focal liver lesions? Med Ultrason 16(1):37–40 24567923 10.11152/mu.2014.2066.161.is1rs2

[2] Tang MX, Mulvana H, Gauthier T, Lim AK, Cosgrove DO, Eckersley RJ et al (2011) Quantitative contrast-enhanced ultrasound imaging: a review of sources of variability. Interface Focus 1(4):520–539 22866229 10.1098/rsfs.2011.0026 PMC3262271

[3] Claudon M, Dietrich CF, Choi BI, Cosgrove DO, Kudo M, Nolsøe CP et al (2013) Guidelines and good clinical practice recommendations for Contrast Enhanced Ultrasound (CEUS) in the liver - update 2012: A WFUMB-EFSUMB initiative in cooperation with representatives of AFSUMB, AIUM, ASUM. FLAUS ICUS Ultrasound Med Biol 39(2):187–210 23137926 10.1016/j.ultrasmedbio.2012.09.002

[4] Dietrich CF, Nolsøe CP, Barr RG, Berzigotti A, Burns PN, Cantisani V et al (2020) Guidelines and Good Clinical Practice Recommendations for Contrast-Enhanced Ultrasound (CEUS) in the Liver-Update 2020 WFUMB in Cooperation with EFSUMB, AFSUMB, AIUM, and FLAUS. Ultrasound Med Biol 46(10):2579–2604 32713788 10.1016/j.ultrasmedbio.2020.04.030

[5] Dietrich CF (2008) Comments and illustrations regarding the guidelines and good clinical practice recommendations for contrast-enhanced ultrasound (CEUS)--update 2008. Ultraschallmed 29(Suppl 4):S188-202 10.1055/s-2008-1027799 18833497

[6] Vidili G, De Sio I, D’Onofrio M, Mirk P, Bertolotto M, Schiavone C (2019) SIUMB guidelines and recommendations for the correct use of ultrasound in the management of patients with focal liver disease. J Ultrasound 22(1):41–51 30580390 10.1007/s40477-018-0343-0 PMC6430299

[7] D’Onofrio M, Crosara S, De Robertis R, Canestrini S, Cantisani V, Morana G et al (2014) Malignant focal liver lesions at contrast-enhanced ultrasonography and magnetic resonance with hepatospecific contrast agent. Ultrasound 22(2):91–98 27433201 10.1177/1742271X13513888 PMC4760537

[8] Quaia E, De Paoli L, Angileri R, Cabibbo B, Cova MA (2014) Indeterminate solid hepatic lesions identified on non-diagnostic contrast-enhanced computed tomography: assessment of the additional diagnostic value of contrast-enhanced ultrasound in the non-cirrhotic liver. Eur J Radiol 83(3):456–462 24387826 10.1016/j.ejrad.2013.12.012

[9] Seitz K, Strobel D, Bernatik T, Blank W, Friedrich-Rust M, Herbay A et al (2009) Contrast-enhanced ultrasound (CEUS) for the characterization of focal liver lesions - prospective comparison in clinical practice: CEUS vs. CT (DEGUM multicenter trial). Parts of this manuscript were presented at the Ultrasound Dreiländertreffen 2008, Davos. Ultraschallmed 30(4):383–389 10.1055/s-0028-1109673 19688670

[10] Bertin C, Egels S, Wagner M, Huynh-Charlier I, Vilgrain V, Lucidarme O (2014) Contrast-enhanced ultrasound of focal nodular hyperplasia: a matter of size. Eur Radiol 24(10):2561–2571 24962831 10.1007/s00330-014-3280-0

[11] Roche V, Pigneur F, Tselikas L, Roux M, Baranes L, Djabbari M et al (2015) Differentiation of focal nodular hyperplasia from hepatocellular adenomas with low-mechanical-index contrast-enhanced sonography (CEUS): effect of size on diagnostic confidence. Eur Radiol 25(1):186–195 25120205 10.1007/s00330-014-3363-y

[12] Dietrich CF, Kratzer W, Strobe D, Danse E, Fessl R, Bunk A et al (2006) Assessment of metastatic liver disease in patients with primary extrahepatic tumors by contrast-enhanced sonography versus CT and MRI. World J Gastroenterol 12(11):1699–1705 16586537 10.3748/wjg.v12.i11.1699 PMC4124343

[13] Bhayana D, Kim TK, Jang HJ, Burns PN, Wilson SR (2010) Hypervascular liver masses on contrast-enhanced ultrasound: the importance of washout. AJR Am J Roentgenol 194(4):977–983 20308500 10.2214/AJR.09.3375

[14] Choi JY, Cho HC, Sun M, Kim HC, Sirlin CB (2013) Indeterminate observations (liver imaging reporting and data system category 3) on MRI in the cirrhotic liver: fate and clinical implications. AJR Am J Roentgenol 201(5):993–1001 24147469 10.2214/AJR.12.10007

